# KIBRA is associated with accelerated cognitive decline and hippocampal atrophy in APOE ε4-positive cognitively normal adults with high Aβ-amyloid burden

**DOI:** 10.1038/s41598-018-20513-y

**Published:** 2018-02-01

**Authors:** Tenielle Porter, Samantha C. Burnham, Vincent Doré, Greg Savage, Pierrick Bourgeat, Kimberly Begemann, Lidija Milicic, David Ames, Ashley I. Bush, Paul Maruff, Colin L. Masters, Christopher C. Rowe, Stephanie Rainey-Smith, Ralph N. Martins, David Groth, Giuseppe Verdile, Victor L. Villemagne, Simon M. Laws

**Affiliations:** 1 Collaborative Genomics Group, Centre of Excellence for Alzheimer’s Disease Research and Care, School of Medical and Health Sciences, Edith Cowan University, Joondalup, 6027 Western Australia, Australia; 2 Co-operative Research Centre for Mental Health, Carlton South, 3053 Victoria, Australia; 3 CSIRO Health and Biosecurity, Parkville, 3052 Victoria, Australia; 4 Centre of Excellence for Alzheimer’s Disease Research and Care, School of Medical and Health Sciences, Edith Cowan University, Joondalup, 6027 Western Australia, Australia; 5 eHealth, CSIRO Health and Biosecurity, Herston, 4029 QLD, Australia; 6 Department of Nuclear Medicine and Centre for PET, Austin Health, Heidelberg, 3084 Victoria, Australia; 7 ARC Centre of Excellence in Cognition and its Disorders, Department of Psychology, Macquarie University, North Ryde, 2113 NSW Australia; 8 Academic Unit for Psychiatry of Old Age, St. Vincent’s Health, The University of Melbourne, Kew, 3101 Victoria, Australia; 9 National Ageing Research Institute, Parkville, 3052 Victoria, Australia; 10 The Florey Institute of Neuroscience and Mental Health, The University of Melbourne, Parkville, 3052 Victoria, Australia; 11 CogState Ltd., Melbourne, 3000 Victoria, Australia; 12 Department of Medicine, Austin Health, The University of Melbourne, Heidelberg, 3084 Victoria, Australia; 13 School of Psychiatry and Clinical Neurosciences, University of Western Australia, Crawley, 6009 Western Australia, Australia; 14 School of Biomedical Sciences, Faculty of Health Sciences, Curtin Health Innovation Research Institute, Curtin University, Bentley, 6102 Western Australia, Australia

## Abstract

A single nucleotide polymorphism, rs17070145, in the KIdney and BRAin expressed protein (*KIBRA*) gene has been associated with cognition and hippocampal volume in cognitively normal (CN) individuals. However, the impact of rs17070145 on longitudinal cognitive decline and hippocampal atrophy in CN adults at greatest risk of developing Alzheimer’s disease is unknown. We investigated the impact rs17070145 has on the rate of cognitive decline and hippocampal atrophy over six years in 602 CN adults, with known brain Aβ-amyloid levels and whether there is an interactive effect with *APOE* genotype. We reveal that whilst limited independent effects of *KIBRA* genotype were observed, there was an interaction with *APOE* in CN adults who presented with high Aβ-amyloid levels across study duration. In comparison to *APOE* ε4-ve individuals carrying the rs17070145-T allele, significantly faster rates of cognitive decline (global, *p* = *0*.*006*; verbal episodic memory, *p* = *0*.*004*), and hippocampal atrophy (*p* = *0*.*04*) were observed in individuals who were *APOE* ε4 + ve and did not carry the rs17070145-T allele. The observation of *APOE* effects in only non-carriers of the rs17070145-T allele, in the presence of high Aβ-amyloid suggest that carriers of the rs17070145-T allele are conferred a level of resilience to the detrimental effects of high Aβ-amyloid and *APOE* ε4.

## Introduction

In cognitively normal older individuals, high levels of neocortical amyloid-β (Aβ-amyloid) are associated with subtle but detectable cognitive decline^1^ and hippocampal atrophy^2^. This observation is consistent with models of Alzheimer’s disease (AD) which propose a protracted preclinical phase that could take up to 20 years^3^. This provides a period of opportunity for understanding, and even interfering with, AD pathogenesis and thus the identification of biological factors, or trait characteristics, that themselves can influence AD progression has become of increased importance.

Several genes have been associated with cognitive performance, particularly episodic memory, and hippocampal atrophy. Previous studies have associated genetic polymorphisms, in particular apolipoprotein E (*APOE*) ε2/ε3/ε4 genotype (see review^4,5^) and the non-synonymous rs6265 (Val66Met) SNP in brain derived neurotropic factor (*BDNF*)^6–9^, with altered rates of episodic memory decline and hippocampal atrophy. Decline in measures of episodic memory, modified by genetic variation, have been reported in both the healthy elderly^10^ and those predicted to be in the early stages of AD based on neocortical Aβ-amyloid imaging^6,7,11^. These findings raise the potential that other genetic factors may also moderate the toxic effects of Aβ-amyloid early in AD and contribute to altered rates of cognitive decline and hippocampal atrophy.

One such candidate is the gene encoding the KIdney and BRAin expressed protein (*KIBRA*; sometimes referred to as WW domain-containing protein 1 (*WWC1*))^12^. KIBRA is a cytoplasmic, signal transducer protein expressed mainly in the kidney and brain^13^ and *in vitro* experiments suggest that, through reduction in postsynaptic levels, it mediates tau induced memory loss and disruption of synaptic plasticity^14^. This *in vitro* data is supported through genetic studies that report the association of allelic variation in the *KIBRA* gene with memory performance, hippocampal atrophy and measurable differences in brain activation. Specifically, a substitution of C for T in the 9^th^ intron (rs17070145), was initially identified through a GWAS of verbal episodic memory performance and replicated in two additional independent cohorts^12^. Episodic memory is one of the earliest cognitive domains to decline, with previous studies observing decline 4–8 years prior to executive function and up to 7–10 years prior to other cognitive domains^15–17^.

However, there is a lack of consensus in subsequent studies that attempted to replicate these genetic associations with memory performance. Cross-sectional studies of cognitively normal (CN) older adults, carriage of the rs17070145-T allele has been associated with better performance in episodic memory^18–22^, delayed recall^23–25^ and spatial learning^26^ and increased hippocampal volume^20^ and activity^19,24^. Conversely, several studies have either associated the absence of rs17070145-T with better semantic^27^ and long-term^28^ memory, executive function^29^ and overall cognitive performance^30^ or were unable to show any association of the SNP with cross sectional episodic memory^29,31–33^ and hippocampal volume^31^ or longitudinal decline in episodic memory and hippocampal volume^31^. However, common to all these studies is the lack of inclusion of Aβ-amyloid imaging, which may contribute to the lack of consensus due to the impact of underlying Aβ-amyloid burden on cognition not being considered^1,6,7,11^.

To address this conjecture requires the availability of comprehensive longitudinal data from the prospective cohort studies of AD, such as the Australian Imaging, Biomarkers and Lifestyle (AIBL) Study, which offers the opportunity to retrospectively evaluate candidate biological factors (e.g. genetic variation) to determine the impact on progression of AD related phenotypes, such as cognitive decline and hippocampal atrophy. The AIBL Study has now more than six years of serial cognitive and neuroimaging assessments, including Aβ-amyloid and structural imaging, in a group of CN adults collected at 18-month intervals. Therefore, the aim of this study was to characterize, through reporting on 6-years of longitudinal data, the role of *KIBRA* rs17070145 allelic variation in this highly characterised CN adult sample and examine the extent to which this allelic variation is associated with Aβ-amyloid related cognitive decline and atrophy of the hippocampus. The hypothesis was that CN adults who carry the rs17070145-T allele would show a slower rate of memory decline and hippocampal atrophy than those not carrying this allele, though this relationship would be dependent on the presence of a high brain Aβ-amyloid burden and interact with *APOE* genotype.

## Results

### The effect of *KIBRA* on cognition and hippocampal atrophy in cognitively normal adults

A total of 602 CN older adults, defined through the AIBL battery of clinical and neuropsychological assessments^34^ were included in this study. As shown in Table 1 there were no significant differences or trends between rs17070145 (henceforth referred to simply as *KIBRA*) T carriers and non-T carriers at baseline with respect to demographic variables, premorbid intellect, depressive symptoms, or genotype. In the initial analysis, co-varied for *APOE* ε4 carrier and Aβ-amyloid status (classified by being above (Αβ^*high*^) or below (Αβ^*low*^) Positron Emission Tomography (PET) Aβ-amyloid tracer-specific thresholds) there were no significant differences in the trajectories between T carriers and non-carriers for measures of global cognition or episodic memory amongst CN adults (Supplementary Figure 1, Supplementary Table 1). However, there was a trend towards T-carriers having a mild improvement (0.028 standard deviations (SD)/year) in both global cognition (non-T carriers, −0.025 SD/year; p = 0.051) and verbal episodic memory (non-T carriers, −0.019 SD/year; p = 0.085), likely due to a practice effect. When evaluating the effect of *KIBRA* on hippocampal atrophy in all cases, and co-varying for *APOE* ε4 carrier and Aβ-amyloid status, no significant difference (p = 0.242) was observed between T carriers (−0.017 cm^3^/year), and non-T carriers (−0.026 cm^3^/year) over six years (Supplementary Figure 1, Supplementary Table 1). Further, no significant differences were observed at baseline in any measures of cognition or hippocampal volume.

**Table 1:** Demographic Information.

		Overall n = 602	*KIBRA* T carrier n = 335	*KIBRA* non-T carrier n = 267	p
Age (years)		70.79 (6.55)	70.73 (6.49)	70.72 (6.41)	0.9788
Female (%)		334 (55.48)	188 (56.12)	146 (54.68)	0.7871
Years of Education	**0–8**	48 (8.00)	27 (8.08)	21 (7.89)	0.9419
	**9–12**	222 (37.00)	127 (38.02)	95 (35.71)	
	**13–15**	126 (21.00)	69 (20.66)	57 (21.43)	
	**15+**	204 (34.00)	111 (33.23)	93 (34.96)	
Premorbid IQ (FSIQ)		107.86 (7.23)	107.66 (7.28)	108.14 (7.30)	0.4311
Depressive Symptoms (GDS)		1.05 (1.28)	1.05 (1.35)	1.04 (1.18)	0.9156
*APOE* ε4 carriage (%)		165 (27.97)	84 (25.53)	81 (31.03)	0.1655
High Aβ-amyloid burden (%)		145 (24.09)	76 (22.69)	69 (25.84)	0.4215
MRI (n)		548	301	247	NA

Baseline demographic and clinical characteristics of all imaged cognitively normal adults in the AIBL study, and based on *KIBRA* rs17070145 T carriage (T_T and C_T) and non-carriage (C_C). p values represent statistical significance when comparing T carriage and non-carriage. GDS, Geriatric Depression Scale; FSIQ, Wechsler Adult Intelligence Scale 3^rd^ Edition (WAIS-III) Full Scale Intelligence Quotient.

**Figure 1:**
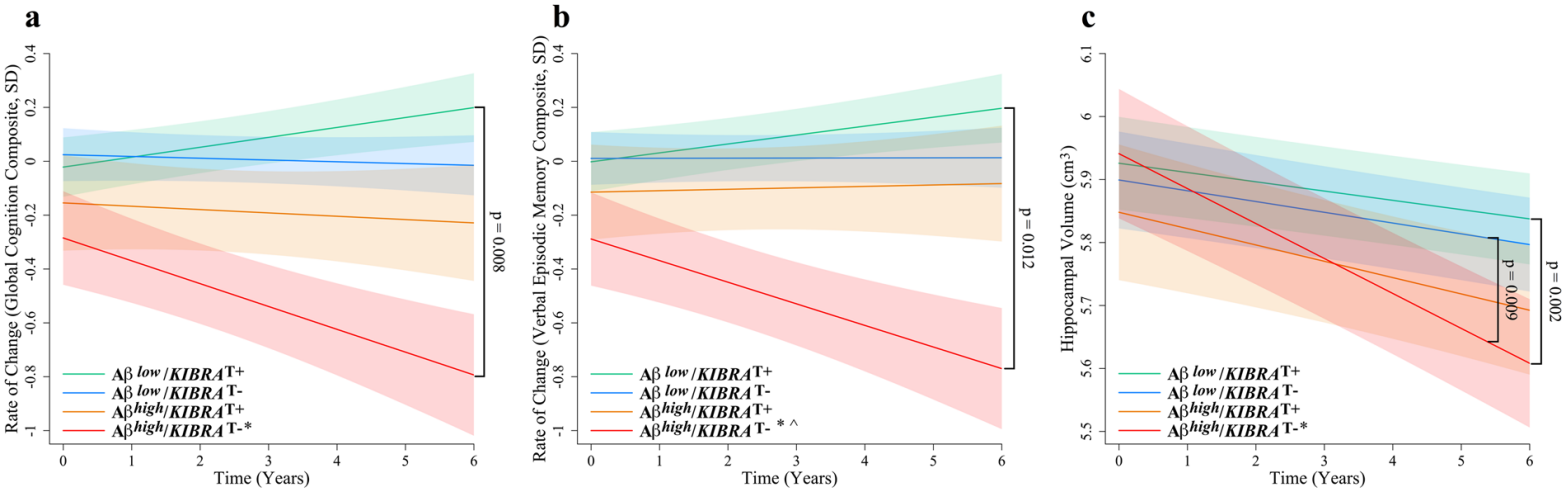
Rates of change in cognitively normal adults based on *KIBRA* T carriage and Aβ-amyloid status. Rates of change are presented for (**a**) a statistically driven global composite, (**b**) a verbal episodic memory composite, and (**c**) hippocampal atrophy (n = 548) in cognitively normal adults (n = 602 unless otherwise stated). Αβ^*low*^, low Αβ-amyloid burden; Αβ^*high*^, high Αβ-amyloid burden. Αβ^*low*^/*KIBRA* T carriers (green), Αβ^*low*^/*KIBRA* non-T carriers (blue), Αβ^*high*^/*KIBRA* T carriers (orange), Αβ^*high*^/*KIBRA* non-T carriers (red), controlling for *APOE* ε4 carrier status. Hippocampal atrophy analysis also controlled for gender (shading represents time dependent standard error, *p < 0.05 when comparing to the Αβ^*low*^/*KIBRA* T carrier group, ^p < 0.05 when comparing to the Αβ^*low*^/*KIBRA* non-T carrier group, ^**φ**^p < 0.05 when comparing to the Αβ^*high*^/*KIBRA* T carrier).

No significant differences were observed at baseline in either measure of cognition or hippocampal volume when investigating the Aβ × *KIBRA* × Time interaction. Relative to Αβ^*low*^/*KIBRA* T carriers, the Αβ^*high*^/*KIBRA* non-T carrier group showed a significantly greater rate of decline in global cognition (0.037 SD/year; −0.085 SD/year; p = 0.008, q = 0.036), and the verbal episodic memory (0.033 SD/year; −0.080 SD/year; p = 0.012, q = 0.042) (Fig. 1, Table 2). However, no statistical difference was seen between Αβ^*high*^/*KIBRA* T carriers and Αβ^*low*^/*KIBRA* non-T carriers. Analysis of hippocampal atrophy revealed that relative to Αβ^*low*^/*KIBRA* T carriers (−0.015 cm^3^/year), the Αβ^*high*^/*KIBRA* non-T carrier group (−0.055 cm^3^/year) showed a significantly greater rate of hippocampal atrophy (p = 0.002, q = 0.034) over six years (Fig. 1, Table 2). Likewise, this trajectory of hippocampal atrophy was also significantly different (p = 0.009, q = 0.034) relative to Αβ^*low*^/*KIBRA* non-T carriers (−0.017 cm^3^/year). In contrast, Αβ^*high*^/*KIBRA* T carriers’ rate of atrophy did not differ from the Αβ^*low*^ groups.

**Table 2:** Group slopes for cognitive composites and hippocampal atrophy in all imaged cognitively normal participants by *KIBRA* carrier and Αβ-amyloid status.

	Αβ^*low*^ *KIBRA* T carrier n = 259	Αβ^*low*^ *KIBRA* non-T carrier n = 198	Αβ^*high*^ *KIBRA* T carrier n = 76	Αβ^*high*^ *KIBRA* non-T carrier n = 69
	β	β	β	β
Global	0.037	−0.006	−0.012	−0.085*
Verbal Episodic Memory	0.033	0.0004	0.005	−0.080*
Hippocampal Atrophy	−0.015	−0.017	−0.026	−0.055*^

Group slopes for cognitive composites (presented in SD/year; n = 602) and hippocampal atrophy (presented in cm^3^/year; n = 548) in all imaged cognitively normal participants, controlling for *APOE* ε4 carrier status. Αβ^*low*^, low Αβ-amyloid burden; Αβ^*high*^, high Αβ-amyloid burden. *p < 0.05 when comparing to the Αβ^*low*^/*KIBRA* T carrier (T_T and C_T) group, ^p < 0.05 when comparing to the Αβ^*low*^/*KIBRA* non-T carrier group, ^**φ**^p < 0.05 when comparing to the Αβ^*high*^/*KIBRA* T carrier.

### The effect of *KIBRA* on cognition and hippocampal atrophy in cognitively normal adults with high Aβ-amyloid

**Figure 2:**
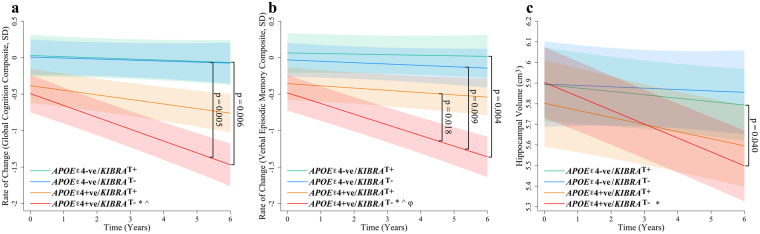
Rates of change in cognitively normal adults with high Aβ-amyloid burden. Rates of change are presented for (**a**) a statistically driven global composite, (**b**) a verbal episodic memory composite, (**c**) hippocampal atrophy in cognitively normal adults with high Aβ-amyloid (n = 145). *APOE* ε4-negative/*KIBRA* T carriers (green), *APOE* ε4-ve/*KIBRA* non-T carriers (blue), *APOE* ε4 + ve/*KIBRA* T carriers (orange), *APOE* ε4 + ve/*KIBRA* non-T carriers (red). Hippocampal atrophy analysis controlled for gender (shading represents time dependent standard error, *p < 0.05 when comparing to the *APOE* ε4-ve/*KIBRA* T carrier group, ^p < 0.05 when comparing to the *APOE* ε4-ve/*KIBRA* non-T carrier group, ^**φ**^p < 0.05 when comparing to the *APOE* ε4 + ve/*KIBRA* T carrier).

No significant differences were observed in Αβ^*high*^ CN adults at baseline in either measure of cognition or hippocampal volume when investigating the *APOE* × *KIBRA* × Time interaction. Relative to *APOE* ε4-ve/*KIBRA* T carriers, the *APOE* ε4 + ve/*KIBRA* non-T carrier group showed a significantly greater rate of decline in global cognition (p = 0.006, q = 0.034) and verbal episodic memory (p = 0.004, q = 0.034) over six years (Fig. 2, Table 3). Further, relative to *APOE* ε4 + ve/*KIBRA* T carriers, the *APOE* ε4 + ve/*KIBRA* non-T carrier group showed a nominally significantly greater rate of decline on the verbal episodic memory composite, however after FDR correction this remained only a strong trend (p = 0.018, q = 0.055) over six years (Fig. 2, Table 3). Hippocampal atrophy analysis revealed that relative to *APOE* ε4-ve/*KIBRA* T carriers (−0.016 cm^3^/year), the *APOE* ε4 + ve/*KIBRA* non-T carrier group (−0.067 cm^3^/year) had nominally significantly different rates of hippocampal atrophy however did not survive correction for multiple testing (p = 0.040, q = 0.107) over six years (Fig. 2, Table 3). This trajectory of hippocampal atrophy was suggestive of being different to *APOE* ε4-ve/*KIBRA* non-T carriers (−0.006 cm^3^/year), however this did not reach significance (p = 0.125), even though this trajectory showed negligible difference to *APOE* ε4-ve/*KIBRA* T carriers. *APOE* ε4 + ve/*KIBRA* T carriers’ rate of atrophy did not differ from the *APOE* ε4-ve groups. To ascertain that these differences in rates of decline were not due to disproportionate rates of clinical conversion, the frequency of individuals who converted to Mild Cognitive Impairment (MCI) or AD over the course of the study was investigated. Within the *APOE* ε4 + ve group there was no significant difference (p = 0.43) between *KIBRA* non-T carriers (0.294, 15 out of 41) and *KIBRA* T carriers (0.294, 10 out of 34) in terms of clinical conversion.

**Table 3:** Group slopes for cognitive composites and hippocampal atrophy in imaged cognitively normal adults with high Aβ-amyloid.

	*APOE* ε4-ve *KIBRA* T carrier n = 38	*APOE* ε4-ve *KIBRA* non-T carrier n = 27	*APOE* ε4 + ve *KIBRA* T carrier n = 34	*APOE* ε4 + ve *KIBRA* non-T carrier n = 40
	β	β	β	β
Global	−0.016	−0.014	−0.063	−0.163*^†
Verbal Episodic Memory	−0.008	−0.019	−0.031	−0.146*^^φ^†
Hippocampal Atrophy	−0.016	−0.006	−0.034	−0.067*

Group slopes for cognitive composites (presented in SD/year) and hippocampal atrophy (presented in cm^3^/year) in imaged cognitively normal adults with high Aβ-amyloid (n = 145). *p < 0.05 when comparing to the *APOE* ε4-ve/*KIBRA* T carrier group, ^p < 0.05 when comparing to the *APOE* ε4-ve/KIBRA non-T carrier group, **φ** p < 0.05 when comparing to the *APOE* ε4 + ve/*KIBRA* T carrier. ^†^q < 0.05 for those reporting nominal significance at p < 0.05.

## Discussion

The data reported here support the hypothesis that *KIBRA* genotype, in combination with *APOE* ε4 and Aβ-amyloid, affects rates of memory decline and hippocampal atrophy in cognitively normal adults. In those CN adults with high Aβ-amyloid burden at baseline, *KIBRA* non-T carriers showed significantly faster decline in the statistically driven global composite, and verbal episodic memory when compared to T carriers with low Aβ-amyloid burden. Within the subset of CN adults with high Aβ-amyloid burden, we showed that those who are *APOE* ε4 + ve and *KIBRA* non-T carriers had significantly faster rates of decline in verbal episodic memory over 6 years, compared to *APOE* ε4 + ve/*KIBRA* T carrier and both *APOE* ε4-ve groups. Importantly, minimal decline was also observed in the *APOE* ε4 + ve/*KIBRA* T carrier group, suggesting that carriage of the *KIBRA* T allele imparts a level of resilience to negative effects of *APOE* ε4 and Aβ-amyloid on memory performance. Further, between group comparisons of the rates of clinical conversion (CN > MCI/AD) over the course of the study revealed no significant differences, suggesting that the faster rates of decline were not due to a higher rate of clinical conversion.

This is further supported by the observations that rates of hippocampal atrophy in this study also differ based on *KIBRA* genotype. In CN adults Aβ-amyloid has been previously reported to be associated with increased hippocampal atrophy^2,35,36^, however in this study this was only observed in those individuals who did not possess the *KIBRA* T-allele, whilst in contrast *KIBRA* T-carriers’ rate of atrophy did not significantly differ from the Αβ^*low*^ groups. In a meta-analysis of *APOE* neuroimaging studies, hippocampal atrophy has been shown to be increased in *APOE* ε4 carriers^5^. Here we report that this association, in a group of Αβ^*high*^ CN individuals, was again only observed in those individuals who did not possess the *KIBRA* T-allele, whilst in contrast *APOE* ε4 + ve/*KIBRA* T-carriers’ rate of atrophy did not differ from the *APOE* ε4-ve groups. Taken together, we propose that the *KIBRA* T allele affords carriers a level of resilience to the detrimental effects of Aβ-amyloid and *APOE* ε4 allele on neurodegeneration, specifically hippocampal atrophy.

The findings presented herein are in line with the original study^12^ and subsequent reports linking the *KIBRA* T allele with resilience in episodic memory performance^18–21,24^. The absence of replication by other studies^27–29,31–33^ may be in part due to the lack of consistency in the measures of memory decline, whereby varying single neuropsychological tests, aiming to measure a certain feature of memory or cognition, were used. The use in this current study of a combination of global and episodic memory composite scores, which encompass several different tests best associated with a cognitive construct, could also have contributed to the ability to detect associations with the *KIBRA* genotype. However, the lack of inclusion of an assessment of underlying Aβ-amyloid burden in the previous studies may in fact be the more telling contributor to the lack of consensus on the association of *KIBRA* with cognitive performance. The level of neocortical Aβ-amyloid is associated with differential rates of cognitive decline^1,37^, and this is further altered by genetic factors, in particular *APOE*^10,11^ and *BDNF*^6,7^. Accounting for the underlying Aβ-amyloid burden in the current study may have further contributed to the detection of differences in rates of cognitive decline and hippocampal atrophy reported with *APOE* ε4 and *KIBRA*.

Whilst the incorporation of cognitive composites and accounting for underlying Aβ-amyloid burden is considered a strength of this study, the following limitations of the study are acknowledged. Firstly, the use of different cognitive tests individually or in combination for the calculation of domain composites, then those specifically described in this study and using the methodology described herein, may yield different results. Second, this study included 6-years of longitudinal follow-up and validation in other longitudinal cohorts, not undertaken herein, over longer durations of follow-up, may result in different findings. Third, the cognitively normal participants in this study were volunteers and not selected at random from the community, they were generally well educated and performed well on cognitive assessments and as such the findings presented herein may be applicable only to similar cohorts. Fourth, there is an overlap between those who are Aβ^*high*^  and those who are *APOE* ε4 + ve, which could confound the results when looking at their interaction. Finally, the *KIBRA* T-allele’s previously reported association with altered brain activation using fMRI^12,19^ could not be tested due to the lack of fMRI data, under a non-resting state, in the AIBL Study.

Studies have previously demonstrated the main areas of *KIBRA* expression in the brain are those also that are implicated in memory function, the hippocampus and temporal cortex^12,38^. Furthermore, increased *KIBRA* gene expression in the temporal cortex^39^ and hippocampus^22^ has been associated with late onset AD. However, in a recent post-mortem brain transcriptomic study in neuropathogically normal individuals by Piras and colleagues a trend towards increased *KIBRA* gene expression was observed in *KIBRA* T homozygotes^40^. Further quantitative PCR analysis reported an over-expression in T-homozygotes compared to C-homozygotes in the hippocampus^40^. Further, the transcriptomic analysis revealed differential activation of the MAPK pathway^40^, a pathway important in learning and memory processes, suggesting a potential mechanism underpinning a decline in memory performance reported in this study. It has also been shown that there is increased hippocampal activity in episodic memory performance tasks in *KIBRA* T carriers when compared with non-T carriers^19^, consistent with the notion of protection from memory decline. *KIBRA* T allele carriers have also been shown to have a decreased levels of brain activation compared to non-T allele carriers in several hippocampal regions activated during memory retrieval^12^. The authors hypothesised that individuals who do not carry the T allele require a greater level of hippocampal activation for memory retrieval^12^.

In addition to the association studies described above, recent *in vivo* evidence provides molecular insights into mechanisms by which *KIBRA* is involved in memory performance. Synaptic plasticity, which is altered in AD, is modulated by dendrin, which in turn binds to the protein that *KIBRA* encodes (KIBRA; see review^41^). Further, KIBRA protein contains a protein kinase C (isoform ζ; PKCζ) binding domain^42^ and has been reported to co-localise with protein kinase M (isoform ζ; PKMζ)^43^, a brain specific variant of PKCζ, which plays important roles in memory formation and long-term potentiation. Johannsen *et al*. have shown the function of the KIBRA protein to be regulated by its C2 domain^38^, which is required for Ca^2+^ binding and is therefore involved in signal transduction in the neurons. This regulation is hypothesised to mediate the effect of the KIBRA protein on memory formation^38^. In a recent study, Tracy and colleagues have proposed a novel mechanism by which acetylated *tau* associated memory loss and disruption of synaptic plasticity is mediated by a reduction in postsynaptic KIBRA protein^14^. This finding links the previous reports of reduced *KIBRA* gene expression in AD with a biological mechanism mediated by acetylated *tau*. Whether the *KIBRA* T allele affords a level of resilience to this loss of synaptic plasticity remains to be determined.

Our findings indicate that *KIBRA* rs17070145 genotype, when combined with high brain Aβ-amyloid burden and *APOE* ε4 carriage, modifies longitudinal rates of decline in verbal episodic memory, a global cognitive composite and hippocampal volume. We propose that early in the disease process of AD, carriers of the *KIBRA* T-allele are conferred a level of resilience to Aβ-amyloid and *APOE* ε4 driven decline. The potential mechanisms by which *KIBRA* contributes to synaptic plasticity, and AD progression warrant further investigation, including the potential impact on Aβ-amyloid accumulation, and may reveal novel pathways contributing to neuroprotection/neurodegeneration. Our results also highlight the potential application of genetics for risk stratification when designing clinical trials, particularly those that employ Aβ-amyloid imaging for screening. The nature of the effects of genetic variations, specifically assessing the combined effect(s) of additional genes affecting cognitive performance would have merit in such settings and requires further investigation.

## Methods

### Participants

This study included 602 CN Caucasian adults enrolled in the AIBL Study, a prospective longitudinal study of ageing. Information regarding the AIBL Study’s design, enrolment process, neuropsychological assessments, and diagnostic criteria has been previously described^34^. The clinical classification of CN, MCI or AD was determined, after clinical review, by a panel of old age psychiatrists, geriatricians, neurologists, and neuropsychologists who were blinded to Aβ-amyloid status. Individuals were classified as CN if they did not meet the clinical criteria for diagnosis of MCI^44^ or dementia^45^, as described previously^34^. Approval of the AIBL Study has been granted by each of the ethics committees of each of the member institutions; Austin Health, St Vincent’s Health, Hollywood Private Hospital, and Edith Cowan University, and informed written consent was given by all volunteers. All clinical investigations were conducted in accord with the principles expressed in the Declaration of Helsinki 1975. All participants were assessed every 18-months. Cognitive, neuroimaging and laboratory assessment were acquired within 3-months of each other.

### Cognitive Measures

The neuropsychological test battery administered in the AIBL study has been described in detail previously^34^. Briefly, it incorporates at each 18-month follow-up, the Mini-Mental State Examination (MMSE), Clock Drawing Test, California Verbal Learning Test-Second edition (CVLT-II), Logical Memory I and II (LMI; LMII; Story A only), D-KEFS verbal fluency, a 30-item version of the Boston Naming Test (BNT), Wechsler Test of Adult Reading (WTAR) for premorbid IQ, Digit Span and Digit Symbol-Coding subtests of the Wechsler Adult Intelligence Scale-Third edition (WAIS-III), the Stroop task (Victoria version), and the Rey Complex Figure Test (RCFT). Resultant data from this battery, in addition to the Clinical Dementia Rating (CDR), have been previously used to statistically derive cognitive composites as previously described^46^. In this study, a verbal episodic memory composite (CDR sum of boxes (CDR_SB_), LMII, CVLT false positives (CVLT_FP_) and long delay free recall (CVLT_LDFR_)), and a statistically driven global composite (CDR_SB_, MMSE, LMII, CVLT_FP_ and Clock), aimed as a sensitive measure for longitudinal decline in individuals predisposed to AD^46^, were investigated across five study time points: baseline, 18, 36, 54 and 72 months. A correction for age, gender, years of education, WTAR-estimated premorbid IQ (WAIS-III Full Scale Intelligence Quotient (FSIQ)) and depressive symptoms (Geriatric Depression Scale (GDS)) was incorporated in the calculation of the cognitive composites^47^.

### Brain Imaging

The 602 CN adults included in this study had undergone Aβ-amyloid imaging, at varying time points, with PET using ^11^C-Pittsburgh Compound B (PiB), ^18^F-florbetapir or ^18^F-flutemetamol as previously described^48–50^. PET standardized uptake value (SUV) ratio (SUVR) data was determined for all tracers using using CapAIBL, a web based freely availably MR-less methodology^51^. Briefly, SUVs were summed and normalized to either the cerebellar cortex SUV (PiB), whole cerebellum SUV (florbetapir) or pons SUV (flutemetamol) to yield the target-region to reference-region SUVR. These SUVRs were then classified as either low (Αβ^*low*^) or high (Αβ^*high*^) Aβ-amyloid burden, based on a tracer-specific SUVR threshold; ≥ 1.5, ≥ 1.10 and ≥ 0.62 for PiB, florbetapir and flutemetamol, respectively, as previously described^52^. Of these 602 participants, 548 also underwent clinical magnetic resonance imaging (MRI) for clinical screening and co-registration with PET images. MRI parameters have been described in detail previously^53^. Briefly, a 3 T T1-weighted MRI was performed using the ADNI magnetization-prepared rapid gradient echo protocol, with an in-plane resolution of 1 × 1 mm and a slice thickness of 1.2 mm. Hippocampal volume was calculated after correcting for age in years and intracranial volume, defined as the sum of grey matter, white matter and cerebrospinal fluid volumes, as previously described^35^.

### Genotyping

DNA extraction from 5 mL of whole blood was performed using QIAamp DNA Blood Maxi Kits (Qiagen, Hilden, Germany) according to manufacturer’s instructions. TaqMan® genotyping assays were used to determine *APOE* (rs7412, assay ID: C____904973_10; rs429358, assay ID: C___3084793_20) and *KIBRA* (rs17070145, assay ID: C__33286269_10) genotypes (Life Technologies, Carlsbad, CA). All TaqMan® genotyping assays were performed on a QuantStudio 12 K Flex™ Real-Time-PCR systems (Applied Biosystems, Foster City, CA) using the TaqMan® GTXpress™ Master Mix (Life Technologies) methodology as per manufacturer instructions. *KIBRA* genotype was observed not depart from Hardy-Weinberg equilibrium. For the purpose of this study *APOE* carrier status is defined by the presence (1 or 2 copies) or absence (0 copies) of the *APOE* ε4 allele, henceforth referred to as *APOE* ε4 + ve or *APOE* ε4-ve, respectively.

### Statistical Analyses

All statistical analyses were performed using Rstudio (Rstudio Team 2015) Version 0.98.1103 for Macintosh^54^. All analyses were performed based on a dominant model for the *KIBRA* rs17070145-T (minor) allele, i.e. T carrier (i.e. C_T and T_T) compared with non-T carrier (i.e. C_C), as per previous studies^12,18–21,24^. Baseline demographic data analyses provided means, standard deviations, and percentages across the entire PET imaged cognitively normal sample and stratified by *KIBRA* rs17070145-T allele carrier (*KIBRA-*T) and non-carrier (*KIBRA* non-T) status. ANOVA (age, premorbid IQ, depressive symptoms) and chi-squared tests (gender, years of education, *APOE* ε4 + ve, high Aβ-amyloid burden) were used to determine the significance of differences between allelic groups. To determine differences in rates of cognitive change and hippocampal atrophy random intercepts linear mixed-effects (LME) models were performed using the “nlme” package in R. LMEs were performed due to their ability to model fixed and random effects, and their robustness when dealing with missing data^55^.

After the inclusion of main effects within the model, i.e. *KIBRA* genotype, interaction terms and covariates were included and modelled as described here. Specifically, to investigate the effect of *KIBRA* on the rate of cognitive decline and hippocampal atrophy, initially a *KIBRA* × Time interaction was modelled across the entire sample, covarying for *APOE* ε4 carrier and Aβ-amyloid status, with the cognitive composites and hippocampal volume as the dependent variables. The effect of Aβ status in combination with *KIBRA* was then investigated by separately modelling an Aβ × *KIBRA* × Time interaction, co-varying for *APOE* ε4 carrier status. The third analysis focused on only Αβ^*high*^ participants, with *APOE* included within an *APOE* × *KIBRA* × Time interaction. In addition, all analyses for hippocampal atrophy co-varied for gender. Graphical representations of all models are presented with time dependent standard error. Further, for all analyses correction for the False Discovery Rate (FDR) using Q-Value (bootstrap method) was performed^56^. Finally, chi-squared analyses were performed between groups to ascertain that group differences in rates of decline were not due to disproportionate rates of clinical conversion over the course of the study.

### Data availability

All data and samples used in this study are derived from the Australian Imaging, Biomarkers and Lifestyle (AIBL) Study of Ageing. All AIBL data, and that specific to this study, is publically accessible to all interested parties through an Expression of Interest procedure and is governed by the AIBL Data Use Agreement, for more information please see https://aibl.csiro.au/awd/.

## Electronic supplementary material

Supplementary material
