# Supplementary material for: KIBRA is associated with accelerated cognitive decline and hippocampal atrophy in APOE ε4-positive cognitively normal adults with high Aβ-amyloid burden
Source: Sci Rep. 2018 Feb 1;8:2034. doi: 10.1038/s41598-018-20513-y (PMC5794989; doi:10.1038/s41598-018-20513-y)
Supplement: Supplementary file 1 — Supplementary material [file 41598_2018_20513_MOESM1_ESM.pdf]

Supplementary Material

KIBRA is associated with accelerated cognitive decline and hippocampal atrophy in APOE ε4-positive cognitively normal adults with high Aβ-amyloid burden

Tenielle Porter[1,2], Samantha C. Burnham[3], Vincent Doré[4,5], Greg Savage[6], Pierrick Bourgeat[4], Kimberly Begemann[1], Lidija Milicic[1], David Ames[7,8], Ashley I. Bush[9], Paul Maruff[9,10], Colin L. Masters[9], Christopher C. Rowe[5,11], Stephanie Rainey-Smith[12], Ralph N. Martins[12,13], David Groth[14], Giuseppe Verdile[12,14], Victor L. Villemagne[5,9,11], and Simon M. Laws[1,2,14]*,

Supplementary Table 1. Mean slopes for cognitive composites and hippocampal atrophy in all imaged cognitively normal participants by *KIBRA* carrier status

	***KIBRA* T carrier**	***KIBRA* non-T carrier**	
	**β**	**β**	**p**
**Global**	0.028	-0.025	0.051
**Verbal Episodic Memory**	0.028	-0.019	0.085
**Hippocampal Atrophy**	-0.017	-0.026	0.242

Mean slopes for cognitive composites (presented in SD/year; n=602) and hippocampal atrophy (presented in cm^3/year; n=548) in all imaged cognitively normal participants, controlling for *APOE* ε4 carrier and Aβ-amyloid status. *Represents a nominally statistically significant difference in slope of the *KIBRA* non-T carrier (C_C) group when compared to the *KIBRA* T carrier (T_T and C_T) group.

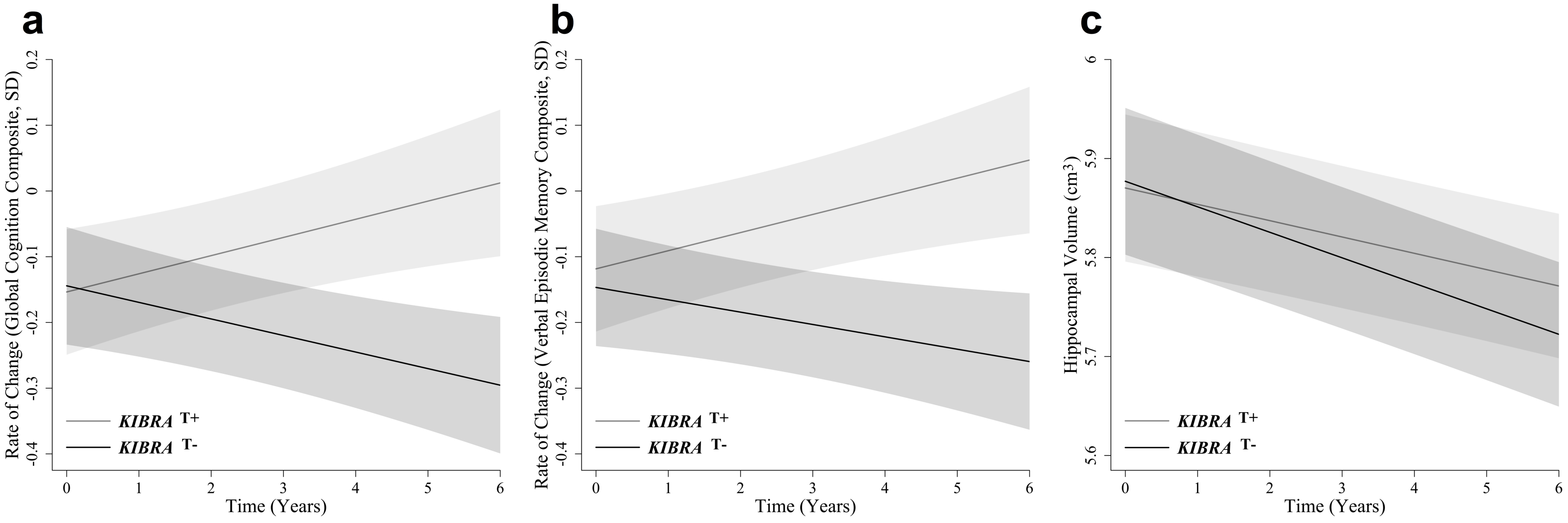

Supplementary Figure 1. Rates of change in cognitively normal adults based on *KIBRA* T carriage.

Rates of change are presented for (a) a statistically driven global composite, (b) a verbal episodic memory composite, (c) hippocampal atrophy (n=548) in cognitively normal adults (n=602 unless otherwise stated). *KIBRA* T carriers (grey) and non-T carriers (black), controlling for *APOE* ε4 carrier and Aβ-amyloid status. Hippocampal atrophy analysis also controlled for gender (shading represents time dependent standard error, * $p<0.05$).
